# The Roles of the Central Executive and Visuospatial Storage in Mental Arithmetic: A Comparison across Strategies

**DOI:** 10.1080/17470218.2013.838590

**Published:** 2014-05-01

**Authors:** Paula J. Hubber, Camilla Gilmore, Lucy Cragg

**Affiliations:** 1 School of Psychology, University of Nottingham, Nottingham, UK; 2 Mathematics Education Centre, Loughborough University, Loughborough, UK

**Keywords:** Visuospatial working memory, Mental arithmetic, Addition, Mathematical cognition, Central executive, Visuospatial sketchpad

## Abstract

Previous research has demonstrated that working memory plays an important role in arithmetic. Different arithmetical strategies rely on working memory to different extents—for example, verbal working memory has been found to be more important for procedural strategies, such as counting and decomposition, than for retrieval strategies. Surprisingly, given the close connection between spatial and mathematical skills, the role of visuospatial working memory has received less attention and is poorly understood. This study used a dual-task methodology to investigate the impact of a dynamic spatial n-back task (Experiment 1) and tasks loading the visuospatial sketchpad and central executive (Experiment 2) on adults’ use of counting, decomposition, and direct retrieval strategies for addition. While Experiment 1 suggested that visuospatial working memory plays an important role in arithmetic, especially when counting, the results of Experiment 2 suggested this was primarily due to the domain-general executive demands of the n-back task. Taken together, these results suggest that maintaining visuospatial information in mind is required when adults solve addition arithmetic problems by any strategy but the role of domain-general executive resources is much greater than that of the visuospatial sketchpad.

Solving arithmetic problems requires a variety of cognitive processes and strategies. For simple sums an answer may be retrieved directly from memory, whilst more complex sums, such as those involving double digits, may require the use of procedural strategies, such as decomposition or counting. Successful execution of these strategies, particularly those of a more procedural nature, has been shown to depend on working memory: the ability to store, monitor, and manipulate information in mind (see De Stefano & LeFevre, 2004; Raghubar, Barnes, & Hecht, 2010, for reviews). Previous research has largely concentrated on the role of verbal working memory in arithmetic. Considerably less attention has been paid to the role of visuospatial working memory, despite considerable evidence of the association between numerical and spatial representations. This is the focus of the current study.

Previous studies have shown the importance of verbal working memory in counting and retaining information whilst doing arithmetic (Bull, Espy, & Wiebe, 2008; Hecht, 2002; Imbo & LeFevre, 2010; Lee & Kang, 2002; Logie, Gilhooly, & Wynn, 1994; Trbovich & LeFevre, 2003). It is thought that verbal working memory resources are used to passively store intermediate values, as well as actively manipulate numerical information when performing carrying operations (Fürst & Hitch, 2000; Logie et al., 1994). These working memory components have been found to play a greater role in procedural than retrieval strategies. Hecht (2002) found that loading verbal working memory with a random letter generation or articulatory suppression task slowed participants’ responses when counting was used to verify an addition problem but not when direct retrieval was used. However, participants use different strategies and processes to solve a verification task from those that they use to generate an answer to an arithmetical problem (Campbell & Tarling, 1996), and therefore this methodology does not accurately assess the contribution of working memory skills to mathematical calculation. Imbo and Vandierendonck (2007) investigated the role of verbal working memory in mathematical calculation by asking participants to listen to Swedish (passive verbal task), or retain and repeat letter strings (active verbal task) while solving single digit addition and subtraction problems using a range of strategies. The active verbal task interfered with counting and decomposition, but not retrieval strategies. This supports previous findings that verbal working memory plays a larger role in procedural than in retrieval strategies.

There is mixed evidence concerning the involvement of visuospatial working memory in arithmetic. A number of recent investigations have found evidence in support of links between arithmetic and visuospatial working memory performance. For example, behavioural performance as well as associated brain activity on a visuospatial working memory task was correlated with subsequent performance on an arithmetic task (Dumontheil & Klingberg, 2012). Similarly, Simmons, Willis, and Adams (2012) found that visuospatial working memory accounted for unique variance in judgements of symbolic magnitude in young children and suggested that it may be particularly important for written addition problems. Also, Reuhkala (2001) found that visuospatial working memory capacity measures, but not verbal working memory measures, correlated with mathematics performance in children aged 15–16 years. In contrast, Noël, Désert, Aubrun, and Seron (2001) investigated whether visual or phonological similarity between numbers would interfere with addition performance. They found evidence only for effects of phonological similarity and thus concluded that verbal working memory, rather than visuospatial working memory, was used to store intermediate results. Similarly, Logie et al. (1994) found minor involvement of visuospatial working memory in arithmetic, and only when problems were presented visually. Other studies have found roles for both visuospatial and verbal working memory. Heathcote (1994) found that solving multidigit sums was affected by visual and spatial interference, with greater disruption to more difficult problems requiring carry-overs. More errors occurred when problems contained visually similar numbers than with visually dissimilar ones. Heathcote suggested that both verbal and visuospatial working memory are involved in solving multidigit sums, whilst Trbovich and LeFevre (2003) found that the impact of loading these subsystems was dependent on the presentation format, with performance in a vertical presentation condition being worse under visual load than under verbal load. In summary, the role of visuospatial working memory in arithmetic is unclear, and in particular previous studies have failed to explore whether visuospatial working memory is differentially recruited across strategies.

There are several reasons to suppose that visuospatial working memory may play an important role in arithmetic. First, a great deal of research has explored links between mathematics ability and general spatial skills (see Mix & Cheng, 2012, for a review). Strong relationships between spatial and mathematical skills have been found across a wide range of ages and tasks. Second, semantic information in multidigit numbers is spatially coded, and so positional information must be taken into account when dealing with numbers greater than 9. As a result, the links between visuospatial working memory and arithmetic may be particularly strong for multidigit arithmetic. Finally, research into the way that adults and children represent and process numbers highlights the spatial nature of numerical representations (for a review see De Hevia, Vallar, & Girelli, 2008). It has been suggested that numerical magnitude representations are inherently spatial in nature (Dehaene, Bossini, & Giraux, 1993), and, in support of this, several authors have proposed that some individuals spontaneously rely on visuospatial processes when solving arithmetic problems, for example by visualizing the numbers involved (e.g., Seron, Pesenti, Noël, Deloche, & Cornet, 1992). However, some apparent spatial–numerical representational links may in fact arise from positional encoding in verbal working memory (van Dijck & Fias, 2011).

Mirroring the effects on verbal working memory, it is likely that the involvement of visuospatial working memory varies according to the arithmetic strategy employed. Visuospatial representation and processing are likely to be particularly important for counting, which emphasizes the ordinal sequence of numbers. Similarly, decomposition strategies, which involve partitioning, storing, and recombining numbers, are likely to require visuospatial involvement. In contrast, it has been proposed that known addition facts are stored in a verbal code (Dehaene, 1992), and therefore retrieval of facts from memory should not require visuospatial working memory.

As well as strategy use, other factors such as problem size are also likely to influence the extent and nature of working memory involvement. A common feature of mental arithmetic is the problem-size effect, whereby error rates and reaction times increase with problem sizes (e.g., De Rammelaere, Stuyven, & Vandierendonck, 1999; Seyler, Kirk, & Ashcraft, 2003). Previous research has largely concentrated on single-digit arithmetic (LeFevre, DeStefano, Coleman, & Shanahan, 2005), although problems involving double digits are likely to be more dependent on working memory because they often require holding interim sums and carry-overs in working memory (Imbo, Duverne, & Lemaire, 2007). The effect of problem size on strategy has been investigated in single-digit arithmetic (Imbo, Vandierendonck, & Rosseel, 2007), but there have been no systematic investigations of larger problem size effects, or the effect that problem size has on visuospatial working memory recruitment.

To summarize, previous research into the role of working memory in arithmetic has focused primarily on the role of verbal working memory. Much less is known about the role of visuospatial working memory and in particular its involvement in different arithmetical strategies. Here we explore this question in two experiments with adult participants.

## Experiment 1

This experiment investigated the role of visuospatial working memory in the performance of retrieval, decomposition, and counting strategies while adults solved single- and double-digit addition problems. Participants were told which strategy to use in order to enable the investigation of strategy execution rather than strategy selection. Our working memory task was designed so that participants had to continually monitor and manipulate information while they remembered and updated the positions of flashing red boxes. Many studies compare conditions with a working memory load to a control condition where no dual task is required. However, this does not rule out the possibility that it is simply completing a dual task that interferes, rather than specifically the working memory demands. Therefore in addition to a no-load condition, our study also included a control task designed to be as similar as possible to the working memory task but without the working memory demands. Each participant completed no load, control load, and working memory load conditions for each of the three strategies (retrieval, decomposition, and counting).

It was hypothesized that visuospatial working memory load would have a significant effect on reaction times, with responses fastest and most accurate in the no load condition and slowest and least accurate for the working memory condition. An interaction was predicted between strategy type and working memory load, with direct retrieval being affected less than procedural strategies when visuospatial working memory was loaded. It was predicted that these effects would be more apparent for problems that involved double digits as these are more reliant on working memory resources.

### Method

#### Participants

Thirty-five participants were recruited from the general population (*M* = 43.2 years, *SD* = 12.1 years, 12 male). No payments were made to participants.

#### Equipment and materials

A Samsung P510 laptop, running Windows XP and E-prime Version 1, was used to present stimuli and record latencies and accuracy. Responses to the addition problems were made using a USB numeric keypad, whilst responses to the secondary visuospatial working memory task were made using the laptop's in-built mouse. Participants used their right hand to use the keypad and left hand to use the mouse.

##### Addition task

Participants were required to answer arithmetic problems using three different strategies: retrieval, counting, and decomposition. For example, for 7 + 6 = : Retrieval—give answer directly from memory; counting—from 7, count upwards 6 times; decomposition—first, add 3 onto 7 to get to 10, then add remaining units to get to the answer. Each problem contained two numbers and was presented horizontally, with the larger number on the left (e.g., 12 + 6 =). Nine sets of 20 experimental problems were used, resulting in 180 experimental problems. Participants were also given eight practice trials for each strategy. Within each problem set, half of the problems comprised solely single digits (1 to 9 omitting 0), and half comprised a double-digit number (max 29) on the left and single-digit number on the right. The averages for sum totals were the same across each problem set. The combination of problem sets with strategy and working memory conditions was counterbalanced. The full set of addition problems can be found in Appendix.

##### Visuospatial task

The visuospatial working memory task was presented at the same time as the arithmetic task and consisted of two rows of four horizontal boxes, with one row above and one row below the presented problems. Different boxes turned red, randomly and one at a time, for 2 seconds, and participants had to respond, using the mouse, when a specified pattern was observed, whilst continuing to answer the addition problems. Three working memory load conditions were used: no load (sum-only task), where the boxes were present on screen, but none turned red, and participants only had to answer the sums; a low-level visuospatial load (zero-back task), where participants had to click the mouse when the box second from left on the top row turned red; and a higher level visuospatial load (two-back task), where participants had to click the mouse when the box that turned red was the same as the box one before last. In other words, a box turned red, and the red then moved to a different box before immediately going back to the box it was just on. For both the zero-back and two-back tasks, an event requiring a response occurred at least on every sixth box turning red. If participants missed an event and did not click the mouse, an auditory “beep” was heard, to remind them to pay attention to the working memory task.

#### Procedure

**Figure 1.**
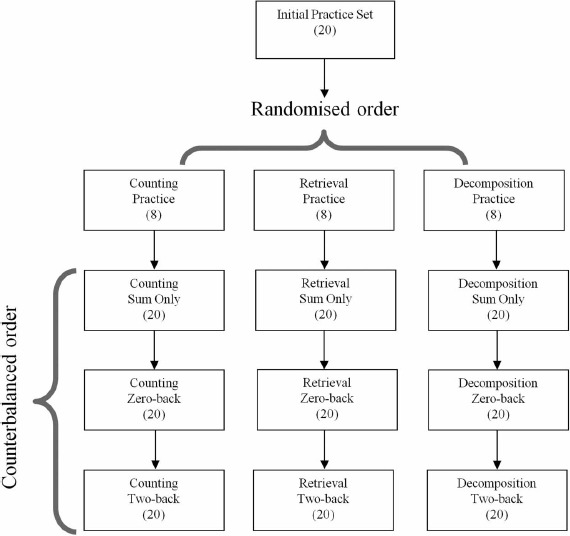
Task structure for Experiment 1. Participants completed all three working memory conditions for a single strategy before moving onto the next strategy.

A within-participants design was used. Participants answered 20 addition problems in each combination of answering strategy and working memory load, giving a total of nine blocks (retrieval with sum-only, zero-back, two-back; decomposition with sum-only, zero-back, two-back; counting with sum-only, zero-back, two-back). The way conditions were presented is depicted in Figure 1.

Participants began by answering a set of 20 practice problems, using a free choice of strategy, before practising the two secondary tasks. They then began the experiment. The order in which the three strategies were used was assigned randomly, and participants completed all three working memory conditions for a single strategy (order counterbalanced) before moving onto the next strategy. Participants were told to give equal attention to the addition problems and the working memory task. All participants were tested individually by the same experimenter, and each session lasted for approximately 30 minutes.

On each trial, the arithmetic problems remained on screen whilst participants worked out the answer using the required strategy. Reaction time was measured from the time the problem appeared until the enter key was pressed. The participant then keyed the answer to the problem using the numeric keypad, before pressing enter again, which triggered the appearance of the next problem. For the zero-back and two-back conditions, the secondary task started when the first problem of each block was presented on the screen and ended when the participant pressed the enter key to make a response on the final problem of the block. The secondary task was paused while participants entered their response but participants were required to remember the previous box location across this delay. At the end of each set of 20 problems, participants were instructed to self-rate, on a scale of 1 to 5, how many of the problems they had used the required strategy for, where 1 was “hardly any” and 5 was “almost all”.

#### Design

Mean accuracy and median reaction times for the arithmetic task, and mean accuracy for the visuospatial tasks, were calculated for each participant. Reaction times (RTs) and accuracy for the arithmetic task were analysed in two separate 3 (strategy: retrieval, decomposition, counting) × 3 (working memory load: sum-only, zero-back, two-back) × 2 (problem size: single digit, double digit) repeated measures analyses of variance (ANOVAs). Mean latencies, mean accuracy, and standard errors are shown in Table 1. Performance on the visuospatial secondary task was also examined by performing a 3 (strategy: retrieval, decomposition, counting) × 2 (working memory load: zero-back, two-back) × 2 (problem size: single digit, double digit) repeated measures ANOVA. Descriptive statistics are shown in Table 2. For all analyses, degrees of freedom were corrected using Greenhouse–Geisser estimates of sphericity where necessary, and post hoc tests were Bonferroni corrected.

**Table 1.** Descriptive statistics for the arithmetic task in Experiment 1

Strategy	Working memory load	RT (ms): Double digit	RT (ms): Single digit	Accuracy: Double digit	Accuracy: Single digit
		*M (SE)*	*M (SE)*	*M (SE)*	*M (SE)*
Retrieval	Sum-only	1463 (147)	1049 (85)	.92 (.02)	.97 (.01)
	Zero-back	1793 (188)	1543 (118)	.92 (.02)	.94 (.01)
	Two-back	2337 (223)	2112 (160)	.88 (.02)	.94 (.01)
Decomposition	Sum-only	3668 (219)	2764 (188)	.92 (.02)	.98 (.01)
	Zero-back	4002 (260)	3416 (223)	.93 (.01)	.94 (.02)
	Two-back	4821 (331)	3897 (320)	.86 (.02)	.93 (.02)
Counting	Sum-only	4214 (228)	2399 (134)	.97 (.01)	.98 (.01)
	Zero-back	4591 (284)	3050 (210)	.93 (.02)	.96 (.01)
	Two-back	5970 (321)	4124 (240)	.87 (.03)	.92 (.02)

*Note*: *M* = mean. *SE* = standard error.

**Table 2.** Descriptive statistics for the visuospatial working memory secondary task in Experiment 1

Strategy	Working memory load	Accuracy: Double digit	Accuracy: Single digit
		*M (SE)*	*M (SE)*
Retrieval	Zero-back	.74 (.06)	.90 (.04)
	Two-back	.54 (.07)	.54 (.07)
Decomposition	Zero-back	.79 (.05)	.79 (.05)
	Two-back	.43 (.04)	.45 (.06)
Counting	Zero-back	.81 (.03)	.85 (.04)
	Two-back	.45 (.04)	.44 (.06)

*Note*: *M* = mean. *SE* = standard error.

### Results

Of the 35 participants, six were removed from the analysis: two participants had a self-rating of “1” at some point on the strategy check, one found the two-back visuospatial task impossible to complete, two struggled to complete conditions containing the zero-back or two-back tasks, and one had reaction times for retrieval that were far slower than their reaction times for decomposition and counting, indicating that they did not follow the retrieval strategy correctly. The remaining participants reported that they had used the required strategies on the majority of trials (retrieval: *M* = 4.94, *SD* = 0.20; decomposition, *M* = 4.69, *SD* = 0.53; counting: *M* = 4.91, *SD* = 0.23).

#### Arithmetic task

##### Reaction times

There was a significant main effect of visuospatial working memory load on RT, *F*(2, 56) = 62.85, *MSE* = 7.34 × 10^7^, *p* < .001. Post hoc tests revealed that all working memory load conditions were significantly different (all *p*s < .001). There was a significant main effect of strategy, *F*(2, 56) = 88.13, *MSE* = 2.83 × 10^8^, *p* < .001. RTs for retrieval were significantly faster than those for decomposition (*p* < .001) and counting (*p* < .001). There was no significant difference for RTs between counting and decomposition (*p* = .456). There was also a significant main effect of problem size, *F*(1, 28) = 116.13, *MSE* = 1.16 × 10^8^, *p* < .001, with slower responses for double-digit than for single-digit problems.

**Figure 2.**
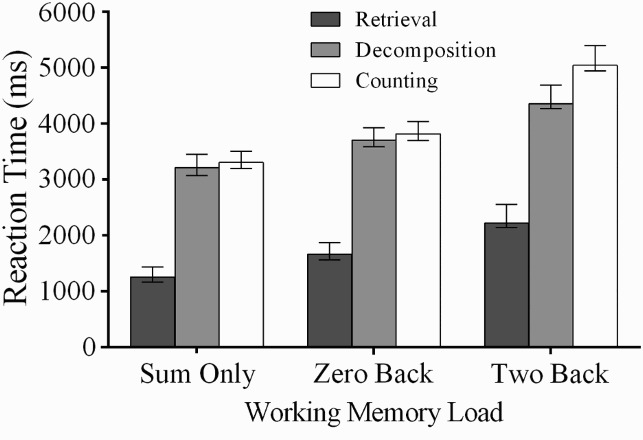
Arithmetic strategy and working memory load interaction for Experiment 1.

A significant interaction was found between working memory load and strategy, *F*(4, 112) = 6.00, *MSE* = 2.88 × 10^6^, *p* < .001, depicted in Figure 2. This indicates that visuospatial working memory load had different effects on RT depending on which arithmetic strategy was used. There was a significant effect of working memory load for each strategy [retrieval, *F*(2, 27) = 26.00, *p* < .001; decomposition, *F*(2, 27) = 19.59, *p* < .001; counting, *F*(2, 27) = 44.54, *p* < .001], with significantly faster RTs in the sum-only condition than in the zero-back condition, and in the zero-back condition than in the two-back condition. However, contrasts revealed that the RT difference between the two-back and zero-back conditions was greater for counting than for retrieval and decomposition (all *p*s < .001). The RT difference between the two-back and zero-back conditions was similar for decomposition and retrieval, *F*(1, 28) = 0.33, *MSE* = 2.54 × 10^5^, *r* = .11. As shown in Figure 2, these contrasts reflect the fact that the harder, two-back visuospatial working memory load (compared to the zero-back task) increased RTs more for the counting strategy than it did for the decomposition and retrieval strategies. There were no significant interactions between strategies when comparing the sum-only condition to the zero-back load condition.

There was also a significant interaction between strategy and problem size, *F*(2, 56) = 93.62, *MSE* = 2.31 × 10^7^, *p* < .001. There was a significant effect of problem size for each strategy [retrieval, *F*(1, 28) = 14.02, *p* < .01; decomposition, *F*(1, 28) = 54.18, *p* < .001; counting, *F*(1, 28) = 185.43, *p* < .001). However, contrasts showed that these effects were not equal. Double digits slowed participants more when using counting than with decomposition, *F*(1, 28) = 63.93, *MSE* = 2.51 × 10^7^, *r* = .83, *p* < .001, and with decomposition than with retrieval, *F*(1, 28) = 33.33, *MSE* = 7.48 × 10^6^, *r* = .74, *p* < .001.

##### Accuracy

There was no significant main effect of strategy on accuracy rates, *F*(2, 56) = 0.98, *MSE* = 0.01, *p* = .384, but there was a significant main effect of working memory load, *F*(1.62, 45.28) = 19.67, *MSE* = 0.17, *p* < .001. Accuracy was significantly higher in the sum-only condition than in the zero-back condition (*p* = .009) and was significantly higher in the zero-back condition than in the two-back condition (*p* = .005). There was also a significant main effect of problem size, *F*(1, 28) = 24.74, *MSE* = 0.22, *p* < .001, with more accurate responses for the single-digit problems.

There was no significant interaction between working memory load and strategy, *F*(2.82, 79.04) = 0.76, *MSE* = 0.01, *p* = .762, but there was a significant interaction between working memory and problem size, *F*(2, 56) = 3.67, *MSE* = 0.02, *p* = .032. Participants were less accurate when the sum contained a double digit for both the sum-only and two-back conditions (sum-only, *p* = .002; two-back, *p* < .001), but not for the zero-back condition (*p =* .101).

#### Visuospatial secondary task

A main effect of working memory load was found, with more accurate performance in the zero-back condition than in the two-back condition *F*(1, 28) = 43.56, *MSE* = 9.87, *p* < .001. Performance on the visuospatial task did not differ across the different arithmetic strategies, *F*(1.58, 44.29) = 1.43, *MSE* = 0.14, *p* = .248, and there was no main effect of problem size, *F*(1, 28) = 2.65, *MSE* = 0.11, *p* = .115, or any significant interactions.

### Discussion

This experiment employed a novel visuospatial working memory load task involving three different load levels combined with counting, decomposition, and retrieval strategies for answering addition problems. The additional effect of problem size was also assessed.

Performance on the arithmetic task was influenced by the visuospatial working memory load. Participants were slower and less accurate in the zero-back condition than in the sum-only condition, and also in the two-back condition than in the zero-back condition. The difference in performance between the sum-only and zero-back conditions supports the inclusion of the zero-back task as a control condition and suggests that some of the effects reported in previous studies, which did not include such a control, may have been due to the general dual-task demands and not the working memory load itself.

Critically, our findings indicate that the impact of a visuospatial working memory load is dependent on the arithmetic strategy used. Counting was slowed more by concurrent visuospatial working memory demands than by decomposition or retrieval. There was no interaction between strategy and load for accuracy, or for secondary task performance, indicating that taxing working memory did not simply lead to differential speed/accuracy or task trade-offs between the three strategies. Moreover, it was not simply driven by the fact that the slowest overall condition showed the largest decrement due to scaling effects, as counting and decomposition strategies were of similar speeds in the sum-only and zero-back conditions.

Although the effects of visuospatial working memory load were greatest for the counting strategy, a significant slowing in response times was also observed when decomposition was used. Interestingly, we also observed a significant effect of the visuospatial working memory load for retrieval. This is surprising because theories of arithmetical cognition predict that retrieval of addition facts should involve verbal, rather than spatial, processes (Dehaene, 1992).

The findings of Experiment 1 suggest a role for visuospatial working memory in arithmetic that is recruited to different extents by different strategies. However, the nature of the visuospatial n-back task used means that it is unclear whether it is the demands of simply holding visuospatial information online, or controlling and manipulating this information, that was interfering with solving the addition problems. According to the Baddeley and Hitch multicomponent model of working memory (Baddeley, 2000, 2003; Baddeley & Hitch, 1974), these two processes rely on different components of working memory: Holding visuospatial information online is the function of the visuospatial sketchpad, which acts as a temporary store for visual and spatial information, whereas controlling and manipulating information in memory is the function of the central executive. This is responsible for attentional control and for the coordination of the visuospatial sketchpad and the phonological loop, a phonological temporary store refreshed through a rehearsal system. The n-back task used in Experiment 1 places a load on both the visuospatial sketchpad and the central executive due to the requirement to continuously monitor and update the box sequence in working memory. Therefore in order to clarify the nature of the interfering working memory demands directly, a second experiment was carried out using standard separate measures of the visuospatial sketchpad and central executive as secondary tasks.

Several researchers have proposed the fractionation of the visuospatial sketchpad, with two subsystems: one, a visual system that holds information such as shape and colour and another that holds information about movement and spatial relations (Baddeley, 2003; Bull, Johnston, & Roy, 1999; Logie et al., 1994). Moreover, Pickering, Gathercole, Hall, and Lloyd (2001) suggested that the visuospatial sketchpad is fractionated between static and dynamic functions, rather than by visual and spatial, as a result of the discovery of a developmental dissociation in performance in the static and dynamic conditions of their experiments. Studies investigating the role of the visuospatial sketchpad in arithmetic have concentrated on loading its static, visual element during dual-task experiments (Imbo & LeFevre, 2010; Lee & Kang, 2002; Trbovich & LeFevre, 2003), such as remembering a pattern of asterisks. However, as suggested here, the dynamic, spatial element of the visuospatial sketchpad also appears to be involved in mental arithmetic (Reuhkala, 2001). Indeed, Hegarty and Kozhevnikov (1999) found that the use of schematic spatial representations, as opposed to pictorial representations, was positively correlated with achievement in mathematical problem solving in 11–13-year-olds. To systematically address the influence of maintaining static and dynamic visuospatial information on mental arithmetic, half of the participants in Experiment 2 completed a visuospatial sketchpad secondary task that involved maintaining static visuospatial information, while the other half completed a dynamic visuospatial sketchpad secondary task. Both groups were also given the same central executive secondary task.

## Experiment 2

### Method

#### Participants

Forty-five undergraduates from the University of Nottingham were recruited and allocated to either the static group (*N* = 22, *M* = 19.8 years, *SD* = 3.2 years, 6 male) or the dynamic group (*N* = 23, *M* = 19.3 years, *SD* = 1.0 years, 5 male) on an alternate basis. Participants received either a course credit or a £6 inconvenience allowance for taking part in the study.

#### Equipment and materials

A Viglen Pentium D computer, running Windows XP and PsychoPy Version 1.73.06 (Peirce, 2007), was used to present stimuli and record latencies and accuracy. Responses to the sums presented were made using a USB numeric keypad, whilst responses to the secondary visuospatial working memory task were made using a mouse. Responses to the central executive secondary task were recorded using a digital sound recorder. Participants used their right hand to use the keypad and their left hand to use the mouse.

##### Addition task

Experiment 2 used the same strategies—retrieval, decomposition, and counting—and the same sets of addition problems as those in Experiment 1.^1^

1In order to ensure that all nine problem sets were matched for mean size of the second addend as well as mean sum total, 8 problems were removed from the analysis leaving a total of 172 experimental trials. This was not required in Experiment 1 because the combination of problem sets with strategy/working memory condition was counterbalanced, something that was not possible in Experiment 2 due to the experimental software.


##### Central executive task

As the central executive is a domain-general resource, a random letter generation task was selected to allow comparison with previous dual-task studies. (e.g., Bull et al., 1999; De Rammelaere et al., 1999; Logie et al., 1994). The requirement to make a letter series random, rather than simply producing a serial string of letters, involves constant attention and switching between retrieval plans, which are controlled by the central executive (Baddeley, 1996). Participants were required to say letters from the alphabet out loud, at random, in time to a metronome set to one beat per second. Letter generation was continuous from the presentation of the first sum to the answering of the final sum in each block. Participants were instructed to avoid strings of letters, such as “a, b, c, d” and were not given a starting letter. Performance on the central executive task was measured by producing a score for randomness of the spoken letters, using RGCalc (Towse & Neil, 1998). The adjacency score measures the percentage of occasions that a spoken letter is directly followed by one of its immediate neighbours in the alphabet.

##### Visuospatial sketchpad task: Static version

Participants were required to memorize the position of four red dots on a 4 × 4 black grid, presented in the centre of the screen. The grid and all four dots on the grid were presented at the same time, for a total of two seconds. Immediately after, an addition problem was presented, which participants had to answer using the required strategy for that block. As soon as the problem had been answered, a blank black grid was presented in the centre of the screen, and participants had to use the mouse to indicate the position of the four red dots, by clicking on the computer screen. The position of the mouse clicks was recorded by PsychoPy (Peirce, 2007). Once the mouse had been clicked four times, the next set of dots to remember was immediately presented. Performance was measured by calculating each participant's proportion correct score for the number of dot positions remembered for each of the three strategies.

##### Visuospatial sketchpad task: Dynamic version

Participants in the dynamic group saw the same grid and sets of dots, but the dots were presented one at a time, for 0.5 seconds each. Once they had answered the problem, participants were required to use the mouse to indicate the position of the dots in the order that they were presented on a blank black grid. Performance was measured by calculating each participant's proportion correct score for the number of dot positions remembered, in the correct order, for each of the three strategies.

#### Procedure

The design was similar to that of Experiment 1. Participants answered 20 addition problems in each combination of answering strategy and working memory load type, giving a total of nine blocks (retrieval with sum-only, visuospatial, central executive; counting with sum-only, visuospatial, central executive; decomposition with sum-only, visuospatial, central executive). Participants began by answering a set of 20 practice problems, using a free choice of strategy, before practising the visuospatial sketchpad and central executive tasks. They then began the experiment. The order in which the three strategies were used was assigned randomly, and participants completed all three working memory conditions for a single strategy (order counterbalanced) before moving onto the next strategy. Participants were told to give equal attention to the addition problems and the working memory tasks. All participants were tested individually by the same experimenter, and each session lasted for approximately 50 minutes.

The addition problems remained on screen whilst participants worked out the answer using the required strategy. Reaction time was measured from the time the problem appeared until the first digit of the answer was pressed. After keying the answer to the problem, the participant pressed enter, which immediately triggered the appearance of the next problem, in the sum-only and central executive conditions, or the grid in the visuospatial condition. As in Experiment 1, at the end of each set of 20 problems, participants were instructed to self-rate on how many of the problems they had used the required strategy to answer, using the numeric keypad, on a scale of 1 to 5, where 1 was “hardly any”, and 5 was “almost all”.

#### Design

Initially, reaction times and accuracy for the arithmetic problems in the visuospatial condition only were analysed in two separate 3 (strategy: retrieval, decomposition, counting) × 2 (problem size: single digit, double digit) × 2 (visuospatial group: static, dynamic) mixed-design ANOVAs, to examine whether the two visuospatial groups performed differently.

**Figure 3.**
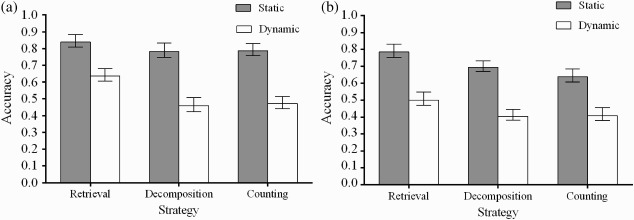
Percentage accuracy for the secondary visuospatial task in Experiment 2 for both dynamic and static groups, whilst answering (a) single-digit and (b) double-digit sums.

Reaction times and accuracy for the full arithmetic task were then analysed in two separate 3 (strategy: retrieval, decomposition, counting) × 3 (working memory type: sum-only, visuospatial, central executive) × 2 (problem size: single digit, double digit) repeated measures ANOVAs. Mean latencies, mean accuracy, and standard errors are shown in Table 3. Accuracy on the secondary tasks was also analysed. For the visuospatial secondary task, a 3 (strategy: retrieval, counting, decomposition) × 2 (problem size: single digit, double digit) mixed ANOVA, with visuospatial task (static, dynamic) as a between-subjects factor, was performed. For the central executive task, a one-way ANOVA was carried out to compare performance for each of the three strategies (retrieval, decomposition, counting). Due to the design of the central executive task, performance could not be compared for single- and double-digit trials separately. Descriptive statistics for secondary tasks are depicted in Figure 3. For all analyses, degrees of freedom were corrected using Greenhouse–Geisser estimates of sphericity where necessary. All post hoc tests were Bonferroni corrected.

**Table 3.** Descriptive statistics for the arithmetic task in Experiment 2

Strategy	Working memory load	RT (ms): Double digit	RT (ms): Single digit	Accuracy: Double digit	Accuracy: Single digit
		*M (SE)*	*M (SE)*	*M (SE)*	*M (SE)*
Retrieval	Sum-only	1556 (84)	1176 (47)	.90 (.02)	.94 (.01)
	Visuospatial	1954 (126)	1683 (113)	.87 (.02)	.94 (.01)
	Central executive	3452 (187)	2662 (150)	.82 (.03)	.89 (.02)
Decomposition	Sum-only	2916 (192)	2656 (183)	.92 (.02)	.94 (.01)
	Visuospatial	3691 (349)	2786 (240)	.94 (.01)	.97 (.01)
	Central executive	6054 (486)	4277 (377)	.90 (.02)	.95 (.01)
Counting	Sum-only	4351 (183)	2358 (125)	.89 (.02)	.97 (.01)
	Visuospatial	4557 (276)	3006 (202)	.93 (.02)	.98 (.01)
	Central executive	9655 (1511)	5833 (883)	.86 (.03)	.95 (.01)

*Note*: *M* = mean. *SE* = standard error.

### Results

Mean accuracy and median reaction times were calculated for each participant. Of the 45 participants, two were removed from the static group (one male, one female) and three from the dynamic group (all female) as they had a self-rating of “1” at some point on the strategy check. The remaining participants reported that they had used the required strategies on the majority of trials (retrieval, *M* = 4.68, *SD* = 0.67; decomposition, *M* = 4.35, *SD* = 0.72; counting, *M* = 4.44, *SD* = 0.62).

#### Comparison of static and dynamic visuospatial groups

There was no main effect of visuospatial group on either RT, *F*(1, 38) < 1, *ns*, or accuracy, *F*(1, 38) = 1.07, *ns*, nor any significant interactions involving visuospatial group. The data were therefore collapsed across group for the analysis of arithmetic task performance.

#### Arithmetic task

##### Reaction times

There was a significant main effect of working memory type on RT, *F*(1.11, 43.34) = 33.30, *MSE* = 9.93 × 10^8^, *p* < .001. Post hoc tests revealed that problems were solved more quickly in the sum-only condition than in the visuospatial condition (*p* = .007), which in turn was faster than the central executive condition (*p* < .001). There was a significant main effect of strategy on RT, *F*(1.33, 51.72) = 34.28, *MSE* = 7.56 × 10^8^, *p* < .001. Problems were solved more quickly using retrieval than using decomposition (*p* < .001), which was faster than counting (*p* = .002). There was also a significant main effect of problem size, *F*(1, 39) = 134.82, *MSE* = 3.07 × 10^8^, *p* < .001, with slower responses for double-digit than for single-digit problems.

**Figure 4.**
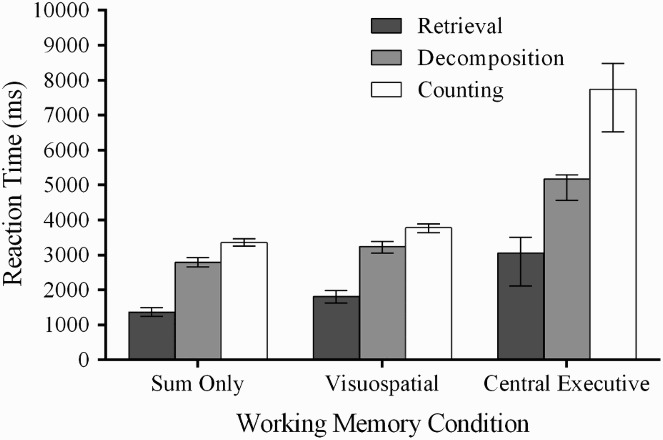
Arithmetic strategy and working memory condition interaction for Experiment 2.

There was a significant interaction between working memory type and strategy, *F*(1.08, 41.91) = 5.71, *MSE* = 1.97 × 10^8^, *p* = 0.019, suggesting that the secondary tasks had different effects on RT depending upon which arithmetic strategy was used. Tests of simple main effects demonstrated that there was a significant effect of working memory load type for each arithmetic strategy [retrieval, *F*(2, 38) = 83.54, *p* < .001; decomposition, *F*(2, 38) = 29.43, *p* < .001; counting, *F*(2, 38) = 7.43, *p* = .002]. For all strategies, problems were solved faster in the sum-only condition than in the visuospatial condition, (*p*s ≤ .05) and faster in the visuospatial condition than in the central executive condition (*p*s < .001). However, contrasts revealed a greater difference between the central executive and visuospatial conditions for counting than for retrieval, *F*(1, 39) = 6.59, *MSE* = 2.97 × 10^8^, *r =* .38, *p* = .014, and decomposition, *F*(1, 39) = 4.92, *MSE* = 1.62 × 10^8^, *r =* .33, *p* = .032, and for decomposition than for retrieval *F*(1, 39) = 7.39, *MSE* = 1.90 × 10^7^, *r =* .40, *p* = .010. As shown in Figure 4, these contrasts reflect the fact that the central executive condition increased RTs more for the counting strategy than it did for the decomposition and retrieval strategies. There was no three-way interaction between strategy, working memory, and problem size, *F*(1.35, 52.45) = 3.31, *MSE* = 1.71 × 10^7^, *p* = .063.

##### Accuracy

There was a significant main effect of working memory load type, *F*(2, 78) = 11.95, *MSE* = 0.13, *p* < .001. Post hoc tests revealed that arithmetic problems were solved more accurately in the sum-only (*p =* .001) and visuospatial (*p* = .001) conditions than in the central executive condition but that there was no difference in accuracy between sum-only and visuospatial (*p* = .82) conditions.

There was also a main effect of strategy, *F*(1.54, 59.98) = 12.52, *MSE* = 0.17, *p* < .001. Post hoc tests revealed that both counting (*p* = .009) and decomposition (*p* < .001) were more accurate than retrieval and that there was no difference in accuracy between counting and decomposition (*p* = .92). A significant main effect of problem size, *F*(1, 39) = 42.54, *MSE* = 0.58, *p* < .001, demonstrated that single-digit sums were solved more accurately than double-digit sums. There were no significant interactions.

#### Central executive secondary task

Mean adjacency scores (standard errors) for the random letter generation task when using each arithmetic strategy were as follows: retrieval, .22 (.02); decomposition, .20 (.01); counting, .21 (.02). There was no main effect of strategy, *F*(1.51, 57.20) = 0.38, *MSE* = 0.00, *p* = .626, showing that participants performed similarly on the central executive task irrespective of which addition strategy they were using.

#### Visuospatial secondary task

There was a main effect of visuospatial task group, with participants in the static group performing significantly more accurately than those in the dynamic group, *F*(1, 38) = 42.71, *MSE* = 4.52, *p* < .001. There was also a significant main effect of strategy, *F*(2, 76) = 24.28, *MSE* = 0.32, *p* < .001. Post hoc tests revealed that performance in the visuospatial task was better whilst using retrieval than whilst using decomposition (*p* < .001) and counting (*p* < .001), but that there was no difference between performance whilst using decomposition and counting (*p* = 1.00). There was also a main effect of problem size, *F*(1, 38) = 40.79, *MSE* = 0.50, *p* < .001, with performance less accurate when answering problems containing double digits.

Although there was a main effect of visuospatial task group, this did not interact with strategy, *F*(2, 76) = 1.51, *MSE* = 0.20, *p* = .227, showing that participants in the dynamic group found the visuospatial task harder than those in the static group, no matter which arithmetic strategy was used. There was no Visuospatial Task × Problem Size interaction, *F*(1, 38) < 1, *ns*. There was, however, a Visuospatial Task × Strategy × Problem Size interaction, *F*(2, 76) = 4.60, *MSE* = 0.04, *p* = .013. As shown in Figure 3, this was driven by a smaller difference in accuracy between the visuospatial task groups when retrieving single digit sums.

### Discussion

Through the inclusion of both central executive and visuospatial load conditions, Experiment 2 enabled us to better understand the findings of Experiment 1. Results showed that the central executive load produced a greater impairment on arithmetic performance than the visuospatial sketchpad load in terms of both slower and less accurate responses. Moreover, the effect of central executive load slowed performance to a greater extent for counting than for decomposition and retrieval, and this was not due to a differential speed/accuracy or task trade-off across strategies. This clarifies the findings of Experiment 1 and indicates that the slowed counting in the two-back condition was likely to be due to increased load on the central executive, rather than the visuospatial nature of the task.

The visuospatial task did not influence accuracy on the arithmetic task compared to the sum-only condition, but it did slow performance, albeit to a lesser extent than the central executive condition. It is not possible to completely rule out that this slowing was due to the general demands of performing a secondary task. However, it appears that maintaining visuospatial information in the visuospatial sketch pad plays a small role in solving addition problems whatever the strategy.

Similar patterns of performance on the arithmetic task were observed for both the static and dynamic visuospatial task groups; however, the dynamic group performed worse on the visuospatial task itself. This may reflect the fact that the dynamic task is more difficult, requiring maintenance of the order as well as location of the stimuli. Better secondary visuospatial task performance for the retrieval strategy may reflect the fact that the visuospatial information did not have to be maintained for as long in this condition.

Experiment 2 also confirmed that working memory load decreases performance more in sums involving double digits than in those only involving single digits, suggesting it plays a greater role in more complex sums. This was true for both visuospatial and central executive load.

## General Discussion

The experiments reported here increase our understanding of the role of working memory in mental arithmetic. Experiment 1 used a dynamic spatial n-back task to explore the role of visuospatial working memory in different arithmetic strategies. The results from this experiment suggested that visuospatial working memory plays a key role in solving arithmetic problems. However, the results of Experiment 2 indicated that it was the central executive demands of monitoring and updating sequences in the n-back task that was critical for mental arithmetic performance, rather than its use of dynamic spatial stimuli. Simply maintaining visuospatial information in mind plays a much lesser role in mental addition.

The present results show the central executive to be involved in counting, decomposition, and retrieval strategies, but to be particularly important for counting. This is consistent with a number of studies demonstrating that procedural strategies rely on the central executive to a greater extent than retrieval strategies (Hecht, 2002; Imbo & Vandierendonck, 2007). The role of the central executive in counting is probably due to the need to store, switch between, and update several different pieces of information. For example, to solve the problem 9 + 4, it is necessary to store the size of the first addend, to increment this total as each counting step is performed (10, 11, 12, 13), and to maintain and update a record of the number of count steps made (1, 2, 3, 4). The coordination of information in memory such as this is known to be a key function of the central executive.

Evidence that cognitive systems that control attention and memory are involved in counting demonstrates that, rather than being a simple strategy, counting can be a complex procedure that is challenging for children. At the early stages of learning mathematics, children rely on counting strategies before progressing, eventually, onto decomposition and retrieval for known facts. However, adults continue to use counting in some situations even when they are given a choice of strategies (Campbell & Austin, 2002). Children who have difficulties with mathematics have been found to rely on counting strategies to a greater extent than children who are proficient with mathematics (e.g., Jordan, Hanich, & Kaplan, 2003), and their counting strategies are more error prone (e.g., Hanich, Jordan, Kaplan, & Dick, 2001). Given the heavy involvement of the central executive, it is possible that it is the domain-general demands of counting that cause difficulties for some children.

The central executive and n-back secondary tasks impaired performance on decomposition strategies, but to a lesser extent than counting. On the one hand this might be surprising because, like counting strategies, decomposition also involves the temporary storage and manipulation of several pieces of numerical information. However, it is possible that some elements of a given decomposition strategy relied on the recall of known facts and thus may have been less reliant on executive processes. Moreover, participants reported using different decomposition methods in the study, including estimating to the nearest 10 then subtracting, adding to the nearest 10, then adding units to get to the answer and also, where the initial addend was double digit, adding the units of the two addends first, before adding the product to the initial decade number. Thus, the use of these somewhat different strategies may have served to mask the overall effects of working memory that were observed. Although the study was designed to investigate strategy execution, there appears to have been an element of strategy selection within the decomposition condition, and this use of different methods should be investigated further, as decomposition strategies may differ in their reliance on working memory resources.

In contrast to previous research (Hecht, 2002; Imbo & Vandierendonck, 2007), this study suggested that even direct retrieval of numerical facts relied on central executive processes to some extent. It is plausible that our use of more difficult two-digit addition problems may have caused participants to use strategies other than retrieval for these problems. However, we found that there was a significant impact of n-back and central executive load for both the single- and double-digit problems. Single-digit addition problems are well learned, and educated adult participants, such as those involved in this study, should be able to directly retrieve these solutions. Retrieval of known facts involves more than just looking up an answer in long-term memory. Although there are some differences among models, it is generally believed that number facts are stored in a network of associations, such that a number pair (e.g., 6 + 7) will be associated with several possible solutions, with differing strengths (Ashcraft, 1992; Campbell, 1995; Siegler & Shrager, 1984). For individuals who are able to retrieve an answer correctly, the correct answer will have the strongest association; however, other surrounding answers may have weaker associations. Therefore in order to retrieve an answer to a known fact, it is necessary to select the appropriate fact and suppress others. In particular it is known that the answers to multiplication facts (i.e., 6 × 7 = 42) will interfere with retrieving the correct answer to known addition facts (i.e., 6 + 7 = 13) and vice versa. It is likely that suppressing incorrect responses will be one process that requires central executive involvement in solving problems by retrieval.

In contrast to the large impact of executive working memory load on mental arithmetic, the visuospatial sketch pad only appeared to play a small role. This contribution was similar across all three strategies, which suggests that the visuospatial sketch pad may have been involved in holding the sum in mind, rather than in performing the different strategies themselves. Given the links between mathematics ability and general spatial skills (e.g., Mix & Cheng, 2012), it is perhaps surprising that there was such a small effect of maintaining visuospatial information on arithmetic performance. This finding contrasts with previous evidence showing relationships between arithmetic performance and visuospatial working memory tasks (Dumontheil & Klingberg, 2012; Heathcote, 1994; Reuhkala, 2001; Simmons et al., 2012; Trbovich & LeFevre, 2003). We see two possible explanations for the limited involvement of visuospatial storage. First, in contrast with previous studies, our participants were well-educated adults rather than children, and we asked them to solve addition problems involving adding a single digit. It is possible that these problems were simple enough for participants to be able to solve them without recourse to visuospatial working memory. Perhaps more complex problems or those involving different operations may have required more visuospatial working memory involvement. Lee and Kang (2002) have suggested that different operations may rely on the use of different working memory subsystems. They found that multiplication slowed with phonological load and subtraction with static visual memory load. Studies involving multiple arithmetical operations and allowing participants to use a wider range of strategies would be needed to better understand the involvement of all components of working memory in arithmetic.

A second possible explanation of the apparent lack of involvement of visuospatial storage is that adult participants have available alternative methods for solving arithmetic problems. So while participants may use visuospatial storage for holding numerical information in some situations, verbal storage may be available as an alternative. Thus when participants are prevented from using visuospatial storage, due to the dual task, they fall back onto using verbal storage. It is possible that there are individual differences between which storage system is the preferred and which is the backup. Similarly, Seron et al. (1992) found that there are wide individual differences in the extent to which participants report visualizing numbers. Contrasting participants’ performance on arithmetic problems with different types of load would be a valuable avenue to explore these possible individual differences.

Aside from debates surrounding the type of storage involved in arithmetic, our results have shown clearly that the working memory system in general is heavily involved in the performance of even simple arithmetic. Research exploring the difficulties that some children have in learning mathematics has tended to focus on the domain-specific problems they have, such as poor representations of number or use of less sophisticated strategies. It is important, however, not to overlook the important role that working memory plays in arithmetic, as deficits in this area may instead underpin the difficulties that some children and adults have with mathematics. Current theories of mathematical cognition tend not to integrate models into a broader system of domain-general cognitive processes and skills. However, it is essential to consider both the domain-specific and domain-general systems together in order to understand the complex interactions between them. For example, individuals may be able to compensate for poor knowledge of mathematical strategies with good working memory capacity, and executive function skills such as inhibition may mediate the relationship between basic numerical representations and mathematics outcomes (Gilmore et al., 2013). There is a need to integrate across research into both the domain-specific and domain-general cognitive systems involved in mathematics performance in order to understand this complex skill and the reasons why many individuals struggle with it.

In summary, we found that the central executive load had a greater impact on the performance of all addition strategies than visuospatial storage load. Counting placed more demands on this aspect of working memory than other strategies, posing particular issues for children and adult learners of arithmetic who tend to rely on this strategy the most. While visuospatial storage load does not appear to be important for mental addition, it may play a role in other types of arithmetic such as subtraction (Lee & Kang, 2002), particularly when the answer is a negative number (Robert & LeFevre, 2013). Future experiments should investigate the effects of central executive and visuospatial load on subtraction, multiplication, and division to gain a fuller understanding of the roles of the different elements of working memory across other types of arithmetic.

## Funding

This project was funded by ESRC (Economic and Social Research Council) project RES-062-23-3280. C.G. is funded by a British Academy Postdoctoral Fellowship.

## Appendix Addition Problems

**Table:**

Retrieval	Counting	Decomposition
Sum Only	Sum Only	Sum Only
1. 6+2	1. 7+2	1. 6+3
2. 5+2	2. 5+2	2. 5+3
3. 5+3	3. 4+2	3. 6+4
4. 5+4	4. 8+4	4. 8+5
5. 6+3	5. 5+3	5. 6+3
6. 7+5	6. 6+2	6. 7+5
7. 9+7	7. 8+7	7. 7+6
8. 8+7	8. 8+5	8. 8+6
9. 8+2	9. 8+4	9. 8+7
10. 9+3	10. 9+5	10. 9+4
11. 13+8	11. 11+8	11. 16+7
12. 14+3	12. 12+7	12. 17+9
13. 19+4	13. 17+6	13. 18+4
14. 21+5	14. 19+9	14. 19+3
15. 23+6	15. 19+7	15. 21+6
16. 24+3	16. 21+8	16. 22+5
17. 25+6	17. 22+7	17. 22+8
18. 25+8	18. 23+6	18. 23+6
19. 27+4	19. 28+8	19. 24+4
20. 28+7	20. 28+9	20. 26+3
Visuospatial	Visuospatial	Visuospatial
1. 6+2	1. 6+2	1. 6+3
2. 5+4	2. 7+2	2. 5+4
3. 7+4	3. 8+3	3. 4+3
4. 4+3	4. 4+3	4. 8+5
5. 5+2	5. 9+5	5. 6+3
6. 8+5	6. 5+3	6. 7+6
7. 6+3	7. 6+3	7. 9+7
8. 7+5	8. 7+4	8. 7+2
9. 9+8	9. 8+2	9. 8+3
10. 8+3	10. 9+7	10. 9+4
11. 12+7	11. 12+6	11. 13+7
12. 17+3	12. 14+5	12. 14+5
13. 18+6	13. 16+5	13. 16+4
14. 19+5	14. 18+7	14. 17+9
15. 21+6	15. 24+4	15. 19+7
16. 22+7	16. 25+4	16. 23+6
17. 26+4	17. 25+9	17. 24+6
18. 27+8	18. 27+6	18. 24+7
19. 27+5	19. 28+7	19. 26+8
20. 29+6	20. 28+6	20. 27+8
Central executive	Central executive	Central executive
1. 6+2	1. 4+3	1. 6+3
2. 4+2	2. 6+3	2. 4+3
3. 9+5	3. 7+2	3. 5+4
4. 5+4	4. 5+4	4. 8+4
5. 6+4	5. 6+5	5. 9+5
6. 6+2	6. 6+2	6. 5+3
7. 8+7	7. 6+4	7. 7+2
8. 7+5	8. 8+5	8. 6+2
9. 9+8	9. 9+7	9. 7+5
10. 9+4	10. 9+3	10. 9+7
11. 13+7	11. 11+4	11. 13+6
12. 15+3	12. 12+5	12. 14+9
13. 16+3	13. 16+8	13. 18+8
14. 18+5	14. 19+7	14. 19+6
15. 19+7	15. 22+7	15. 19+9
16. 21+7	16. 23+5	16. 21+4
17. 23+7	17. 24+7	17. 22+7
18. 28+6	18. 26+6	18. 24+5
19. 28+7	19. 28+9	19. 27+8
20. 29+5	20. 28+8	20. 28+8
